# Oral administration of short chain fatty acids could attenuate fat deposition of pigs

**DOI:** 10.1371/journal.pone.0196867

**Published:** 2018-05-03

**Authors:** A. R. Jiao, H. Diao, B. Yu, J. He, J. Yu, P. Zheng, Z. Q. Huang, Y. H. Luo, J. Q. Luo, X. B. Mao, D. W. Chen

**Affiliations:** Institute of Animal Nutrition, Sichuan Agricultural University, Key Laboratory for Animal Disease-Resistance Nutrition of China Ministry of Education, Ya’an, Sichuan, People’s Republic of China; Southern Illinois University School of Medicine, UNITED STATES

## Abstract

Short chain fatty acids (SCFAs) are the main products of indigestible carbohydrates that are fermented by microbiota in the hindgut. This study was designed to investigate the effects of oral SCFAs administration on the lipid metabolism of weaned pigs. A total of 21 barrows were randomly allocated into three groups, including control group (orally infused with 200 mL physiological saline per day), low dose SCFAs group (orally infused with 200 mL SCFAs containing acetic acid 20.04 mM, propionic acid 7.71 mM and butyric acid 4.89 mM per day), and high dose SCFAs group (orally infused with 200 mL SCFAs containing acetic acid 40.08 mM, propionic acid 15.42 mM and butyric acid 9.78 mM per day). The results showed that the average daily feed intake of SCFAs groups were lower than that of control group (*P*<0.05). Oral administration of SCFAs decreased the concentrations of triglyceride (TG), total cholesterol (TC), high density lipoprotein-cholesterol and insulin (*P*<0.05), and increased the leptin concentration in serum (*P*<0.05). The total fat, as well as TC and TG levels in liver, was decreased by oral SCFAs administration (*P*<0.05). In addition, SCFAs down-regulated the mRNA expressions of fatty acid synthase (FAS) and sterol regulatory element binding protein 1c (*P*<0.05), and enhanced the mRNA expression of carnitine palmitoyltransferase-1α (CPT-1α) in liver (*P*<0.05). SCFAs also decreased FAS, acetyl-CoA carboxylase (ACC) and peroxisome proliferator activated receptor σ mRNA expressions in longissimus dorsi (*P*<0.05). And in abdominal fat, SCFAs reduced FAS and ACC mRNA expressions (*P*<0.05), and increased CPT-1α mRNA expression (*P*<0.05). These results suggested that oral administration of SCFAs could attenuate fat deposition in weaned pigs via reducing lipogenesis and enhancing lipolysis of different tissues.

## Introduction

In recent years, owing to decreased physical activities and increased energy intake, metabolic syndrome, especially obesity, has become a global epidemic [1, 2]. And the incidence of obesity in most western countries is over 20%, which prompts us to find new therapies that are more effective [3]. Nowadays, the role of dietary fiber in body weight management has caught more and more attention [4, 5]. This comes from the fact that increased fermentable fiber intake is beneficial to obesity control through suppressing appetite and modulating energy metabolism [6]. Feeding rodents with dietary fiber inhibited high fat diet-induced body weight gain and fat accumulation [7, 8]. Diets supplemented with fermentable fiber also helped to improve glucose homeostasis in humans [9]. Thus, there have been renewed interests in investigating the mechanism that dietary fiber suppresses appetite and obesity.

The hindgut is colonized by a large amount of microorganisms, which we call microbiota. They can ferment some nondigestible carbohydrates that escape absorption in small intestine, including dietary fiber. And short chain fatty acids (SCFAs), especially acetate, propionate and butyrate, are the main products of bacteria fermentation [10]. SCFAs have been shown to play important roles in shaping gut environment, maintaining electrolyte balance and providing energy for host cells as well as gut microbiota [11]. A growing number of *in vivo* and *in vitro* studies have demonstrated that SCFAs contribute greatly to energy homeostasis and lipid metabolism through stimulating several hormonal and neural signals at multiple tissues [12, 13].

However, the systematic impacts of SCFAs on lipid metabolism have been rarely studied, and the further mechanisms need to be investigated. Importantly, pigs share high similarities with humans regarding genetics, anatomy and physiology, which are considered better models when studying human nutrition and disease compared with rodents. Accordingly, the present study was conducted to investigate the effects of oral SCFAs administration on lipid metabolism in pigs, which could provide some insights into the mechanism that dietary fiber modulated body weight gain and fat accumulation.

## Materials and methods

### Ethics approval and consent to participate

All experimental procedures and animal care were accomplished in accordance with the Guide for the Care and Use of Laboratory Animals provided by the Institutional Animal Care Advisory Committee for Sichuan Agricultural University. All animal protocols used in this study were approved by the Animal Care and Use Committee of Sichuan Agricultural University under permit number DKY-B20131704.

### Animal, management and diet

All experimental procedures and animal care were accomplished in accordance with the Guide for the Care and Use of Laboratory Animals provided by the Institutional Animal Care Advisory Committee for Sichuan Agricultural University. All animal protocols used in this study were approved by the Animal Care and Use Committee of Sichuan Agricultural University under permit number DKY-B20131704. A total of 21 healthy barrows (Duroc×Landrace×Yorkshire) with average initial body weight (8.31 ± 0.06 kg) were randomly allocated into 3 groups (n = 7). The groups were: 1) control group (orally infused with 200 mL physiological saline per day), 2) Low dose SCFAs group (orally infused with 200 mL SCFAs containing acetic acid 20.04 mM, propionic acid 7.71 mM and butyric acid 4.89 mM per day), 3) High dose SCFAs group (orally infused with 200 mL SCFAs containing acetic acid 40.08 mM, propionic acid 15.42 mM and butyric acid 9.78 mM per day). All the pigs were individually penned in metabolism cages (1×0.5×0.8m) under temperature, humidity and light control during the 1 week experimental period. The pH of SCFAs were adjusted to around 6 with sodium hydroxide or hydrochloric acid. Diet was formulated to meet or exceed the nutrient requirement of NRC (2012) recommendation for 7–11 kg pigs, and the compositions were presented in Table 1. Pigs were provided with *ad libitum* access to water and feed during the study.

**Table 1:** Composition and nutrient level of experimental diets (air dry basis %).

Ingredient	Content	Calculated Composition	Nutrient content
Corn	28.79	DE(MJ/kg)	3.55
Extruded corn	27.61	CP	19.59
Dehulled soybean meal	10.33	CF	1.88
Extruded soybean	4.50	Ca	0.81
Fish meal	0.50	TP	0.57
Whey powder	8.00	AP	0.37
Soybean protein concentrate	12.00	Lys	1.36
Soybean oil	1.90	Met+Cys	0.75
Sucrose	3.50	Thr	0.79
Limestone	0.91	Trp	0.23
Dicalcium phosphate	0.74		
Nacl	0.25		
78% Lys	0.38		
*DL*-Met	0.17		
98.5%Thr	0.05		
98%Trp	0.02		
Chloride choline	0.10		
Vitamin premix^1^	0.05		
Mineral premix^2^	0.20		
Total	100.00		

^1^The premix provides following per kg diet: VA 5512 IU, VD_3_2250 IU, VE 24 mg, VK_3_ 3 mg, VB_2_ 6 mg, VB_6_ 3 mg, VB_12_ 24 μg, folic acid 1.2 mg, nicotinic acid 14 mg, biotin 150 μg,*D*-pantothenic acid 15 mg.

^2^The premix provides following per kg diet: Fe100 mg, Cu6 mg, Mn 4 mg, Zn100 mg, I 0.14 mg, Se 0.3 mg.

### Growth performance

The body weight of each pig was measured on the morning of day 1 and 8 before feeding. The feed intake of pigs was recorded each day. These were used to calculate average daily weight gain (ADG), average daily feed intake (ADFI) and the ratio of feed to gain (F/G).

### Slaughter and sample collection

On the morning of day 8, after weighing, blood samples were collected by acute jugular venipuncture, centrifuged at 3000 × g, and stored at -20°C. And then, all pigs were slaughtered according to previously described procedures [14]. Liver, longissimus dorsi and abdominal fat were collected and stored at -80°C for further analyses.

### Biochemical analyses

About 0.6 g frozen liver sample of each pig was homogenized on ice with 5.4 mL physiological saline, and centrifuged at 3000 × g. Then, the supernatant was collected and stored at -20°C for further biochemical analyses. The triglyceride (TG), total cholesterol (TC), high density lipoprotein-cholesterol (HDL-c), low density lipoprotein-cholesterol (LDL-c) and glucose of serum and liver were measured by commercial assay kits from Nanjing Jiancheng Biochemistry (Nanjing, China) according to the manufacturer’s instructions. The insulin, glucagon and leptin were measured by commercial enzyme-linked immunosorbent assay (ELISA) kits from Xinle Co. Ltd. (Shanghai, China) according to the manufacturer’s instructions. Liver total fat was measured according to previously described procedures [15].

### RNA isolation and reverse transcription

Total RNA was isolated from liver, longissimus dorsi and abdominal fat by using Trizol Reagent (TaKaRa Biotechnology, Dalian, China) according to the manufacturer’s protocols. The purity and concentration of total RNA were determined by spectrophotometer detection (Beckman Coulter DU800), and the OD_260_:OD_280_ ratio ranged from 1.8 to 2.0 in all samples. The RNA integrity was analyzed by 1% agarose gel electrophoresis. The RNA samples were reversely transcribed into complementary DNA by using RT Reagents (TaKaRa Biotechnology, Dalian, China) according to the manufacturer’s protocols.

### Real-time quantitative PCR

Following reverse transcription, mRNA levels were analyzed by real-time quantitative PCR using SYBR Premix Ex Taq reagents (TaKaRa Biotechnology, Dalian, China) and CFX-96 Real-Time PCR Detection System (Bio-Rad Laboratories, Richmond, CA) as previously described [16]. The primers were purchased from TaKaRa Biotechnology (Dalian, China), which were shown in Table 2. The PCR condition was as follows: pre-denaturation 95°C for 30 s, followed by 40 cycles at 95°C for 5 s, then at annealing temperature for 30 s, finally at 72°C for 60 s. A melting curve was conducted to verify the specificity. Analysis of each sample was repeated in triplicate simultaneously on the same PCR plate. The average value of each triplicate was used for statistical analysis. The relative mRNA expression to the reference gene (β-actin) was determined in order to correct for the variance in amounts of RNA input in the reaction. In addition, the relative gene expressions compared to the reference gene were calculated with the previous method [17].

**Table 2:** Primer lists used for real time PCR assay.

Gene name	Sequence	
β-actin	Sense	TCTGGCACCACACCTTCT
	Antisense	TGATCTGGGTCATCTTCTCAC
FAS	Sense	CTACGAGGCCATTGTGGACG
	Antisense	AGCCTATCATGCTGTAGCCC
ACC	Sense	AGCAAGGTCGAGACCGAAAG
	Antisense	TAAGACCACCGGCGGATAGA
PPAR-α	Sense	CGACCTGGAAAGCCCGTTAT
	Antisense	GAGGCTTTGTCCCCACAGAT
PPAR-σ	Sense	CTCTTCCTCAACGACCAGGT
	Antisense	GCAGCCCATCCTTATTGACG
PPAR—γ	Sense	CCAGCATTTCCACTCCACACTA
	Antisense	GACACAGGCTCCACTTTGATG
SREBP-1c	Sense	AAGCGGACGGCTCACAATG
	Antisense	GCAAGACGGCGGATTTATTCA
LPL	Sense	CACATTCACCAGAGGGTC
	Antisense	TCATGGGAGCACTTCACG
CPT-1α	Sense	GACAAGTCCTTCACCCTCATCGC
	Antisense	GGGTTTGGTTTGCCCAGACAG
LIPE	Sense	GCCTTTCCTGCAGACCATCT
	Antisense	CACTGGTGAAGAGGGAGCTG

FAS fatty acid synthase; ACC acetyl-CoA carboxylase; PPAR peroxisome proliferator activated receptor; SREBP-1c sterol regulatory element binding protein 1C; LPL lipoprotein lipase; CPT-1α carnitine palmitoyltransferase-1α; LIPE lipase hormone- sensitive

### Statistical analysis

All data were analyzed by one-way ANOVA model followed by Duncan’s multiple-range tests using SPSS 20.0 for Windows statistical software package (Statistical Product and Service Solutions, Inc, USA). Results were presented as the mean and SE. *P*<0.05 were used to assess statistical significance.

## Results

### Growth performance

According to Table 3, during the experimental period, pigs that were orally administered with SCFAs had lower ADFI compared with those in the control group (*P*<0.05). However, no significant differences were observed among the three groups regarding ADG and F/G (*P*>0.05).

**Table 3:** Effects of SCFAs on the growth performance over the experimental period of weaned pigs.

	Control	SCFA (L)	SCFA (H)	SEM	*P*-value
Initial BW (kg)	8.32	8.31	8.31	0.06	0.994
Final BW (kg)	9.33	9.13	9.30	0.27	0.842
ADFI (g/d)	258.10^a^	218.69^b^	231.55^a^^b^	8.21	0.020
ADG (g/d)	173.81	125.00	159.52	13.42	0.071
F/G	1.59	1.76	1.48	0.14	0.389

SCFAs (L): acetic acid 20.04 mM, propionic acid 7.71 mM and butyric acid 4.89 mM; SCFAs (H) :acetic acid 40.08 mM, propionic acid 15.42 mM and butyric acid 9.78 mM.

BW body weight; ADFI average daily feed intake; ADG average daily gain; F/G the ratio of feed to gain

^a-b^Within a row, means without a common superscript differ (*P*<0.05).

### Serum metabolites

As shown in Table 4, oral administration of SCFAs decreased the concentrations of TG, TC and HDL-C in serum (*P*<0.05). Moreover, high dose SCFAs group had lower insulin concentration and greater leptin concentration in serum compared with control group (*P*<0.05). We did not observe any differences in serum LDL-C, glucose and glucagon among the three groups (*P*>0.05).

**Table 4:** Effects of SCFAs on the serum metabolites over the experimental period of weaned pigs.

	Control	SCFA (L)	SCFA (H)	SEM	*P*-value
TG mmol/L	0.56^a^	0.26^b^	0.24^b^	0.05	0.004
TC mmol/L	2.67^a^	1.63^b^	1.64^b^	0.19	0.026
HDL-c mmol/L	1.41^a^	0.88^b^	0.79^b^	0.09	0.004
LDL-c mmol/L	0.58	0.41	0.42	0.05	0.378
Glucose mmol/L	6.99	6.31	6.78	0.23	0.499
Insulin μIU/mL	20.18^a^	14.11^a^^b^	11.35^b^	1.51	0.039
Glucagon μIU/mL	247.83	146.35	99.07	29.28	0.100
Leptin ng/mL	5.66^b^	5.79^b^	7.27^a^	0.27	0.015

SCFAs (L): acetic acid 20.04 mM, propionic acid 7.71 mM and butyric acid 4.89 mM; SCFAs (H) :acetic acid 40.08 mM, propionic acid 15.42 mM and butyric acid 9.78 mM.

TG triglyceride; TC total cholesterol; HDL-c high density lipoprotein-cholesterol; LDL-c low density lipoprotein-cholesterol.

^a-b^Within a row, means without a common superscript differ (*P*<0.05).

### Liver total fat and metabolites

As shown in Table 5, SCFAs treatments had lower total fat of liver than control group (*P*<0.05). Liver TG was also markedly decreased when treated with SCFAs (*P*<0.05). Furthermore, high dose SCFAs group had lower liver TC, compared with the other two groups (*P*<0.05). There were no differences in liver HDL-C, LDL-C, glucose, insulin, glucagon and leptin among the three groups (*P*>0.05).

**Table 5:** Effects of SCFAs on the liver lipids and metabolites over the experimental period of weaned pigs.

	Control	SCFA (L)	SCFA (H)	SEM	*P*-value
Liver total fat (g/100g)	3.34^a^	2.92^b^	2.79^b^	0.08	0.007
TG mmol/L	1.05^a^	0.71^b^	0.71^b^	0.06	0.006
TC mmol/L	0.93^a^	0.83^a^	0.51^b^	0.06	0.008
HDL-c mmol/L	0.33	0.17	0.18	0.05	0.351
LDL-c mmol/L	0.81	0.43	0.30	0.09	0.073
Glucose mmol/L	10.72	9.87	9.35	0.39	0.714
Insulin μIU/mL	31.01	29.63	29.73	0.77	0.743
Glucagon μIU/mL	767.00	750.73	656.00	22.75	0.092
Leptin ng/mL	6.12	6.10	6.11	0.08	0.993

SCFAs (L): acetic acid 20.04 mM, propionic acid 7.71 mM and butyric acid 4.89 mM; SCFAs (H) :acetic acid 40.08 mM, propionic acid 15.42 mM and butyric acid 9.78 mM.

TG triglyceride; TC total cholesterol; HDL-c high density lipoprotein-cholesterol; LDL-c low density lipoprotein-cholesterol.

^a-b^Within a row, means without a common superscript differ (*P*<0.05).

### The expressions of lipid metabolism related genes in liver, longissimus dorsi and abdominal fat

**Fig 1:**
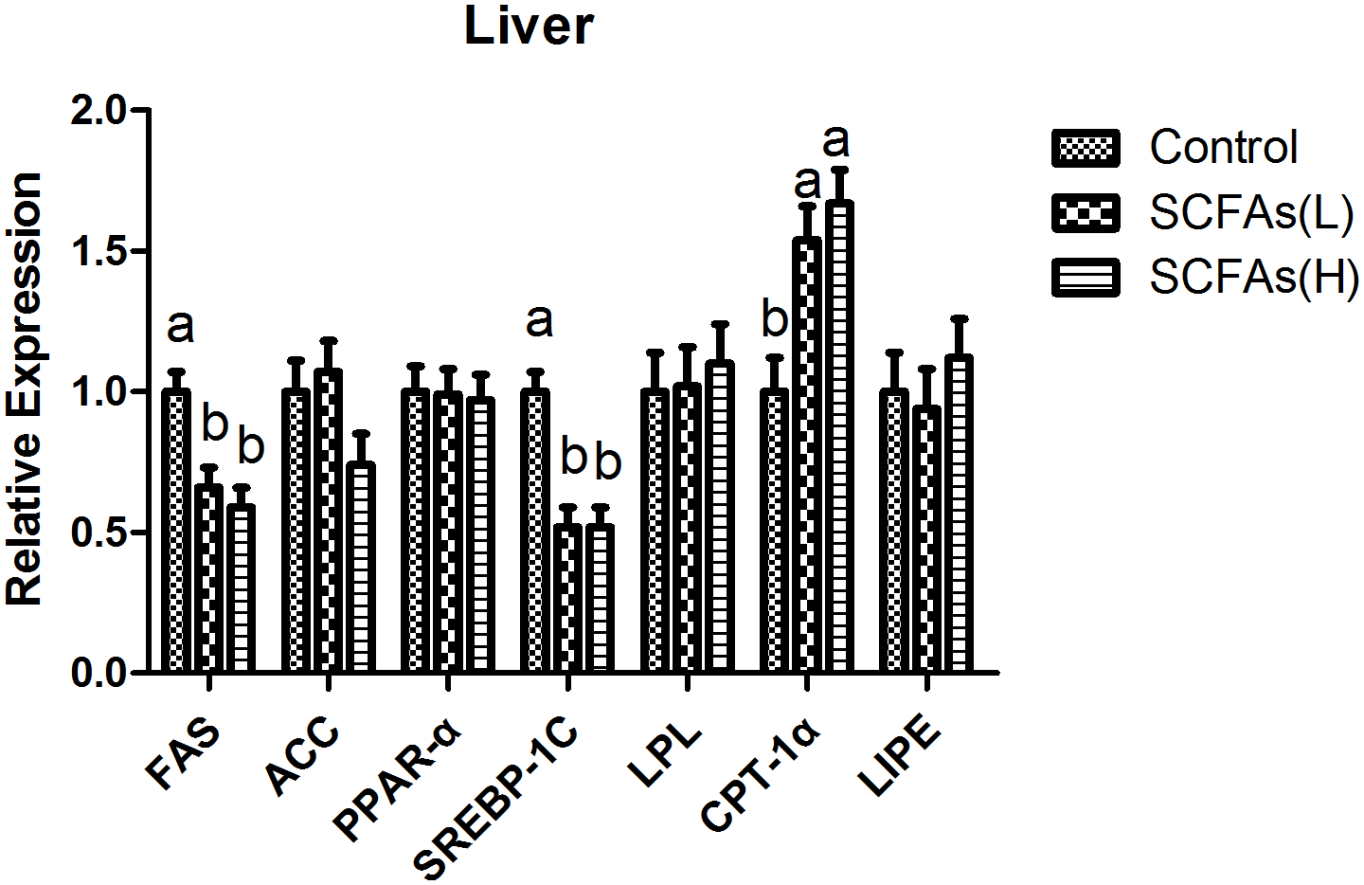
Effects of SCFAs on mRNA expressions for key factors related to lipid metabolism in liver over the experimental period of weaned pigs. SCFAs (L): acetic acid 20.04 mM, propionic acid 7.71 mM and butyric acid 4.89 mM; SCFAs (H) :acetic acid 40.08 mM, propionic acid 15.42 mM and butyric acid 9.78 mM. FAS fatty acid synthase; ACC acetyl-CoA carboxylase; PPAR peroxisome proliferator activated receptor; SREBP-1C sterol regulatory element binding protein 1C; LPL lipoprotein lipase; CPT-1α carnitine palmitoyltransferase-1α; LIPE lipase hormone- sensitive. ^a-b^Within a row, means without a common superscript differ (P<0.05).

**Fig 2:**
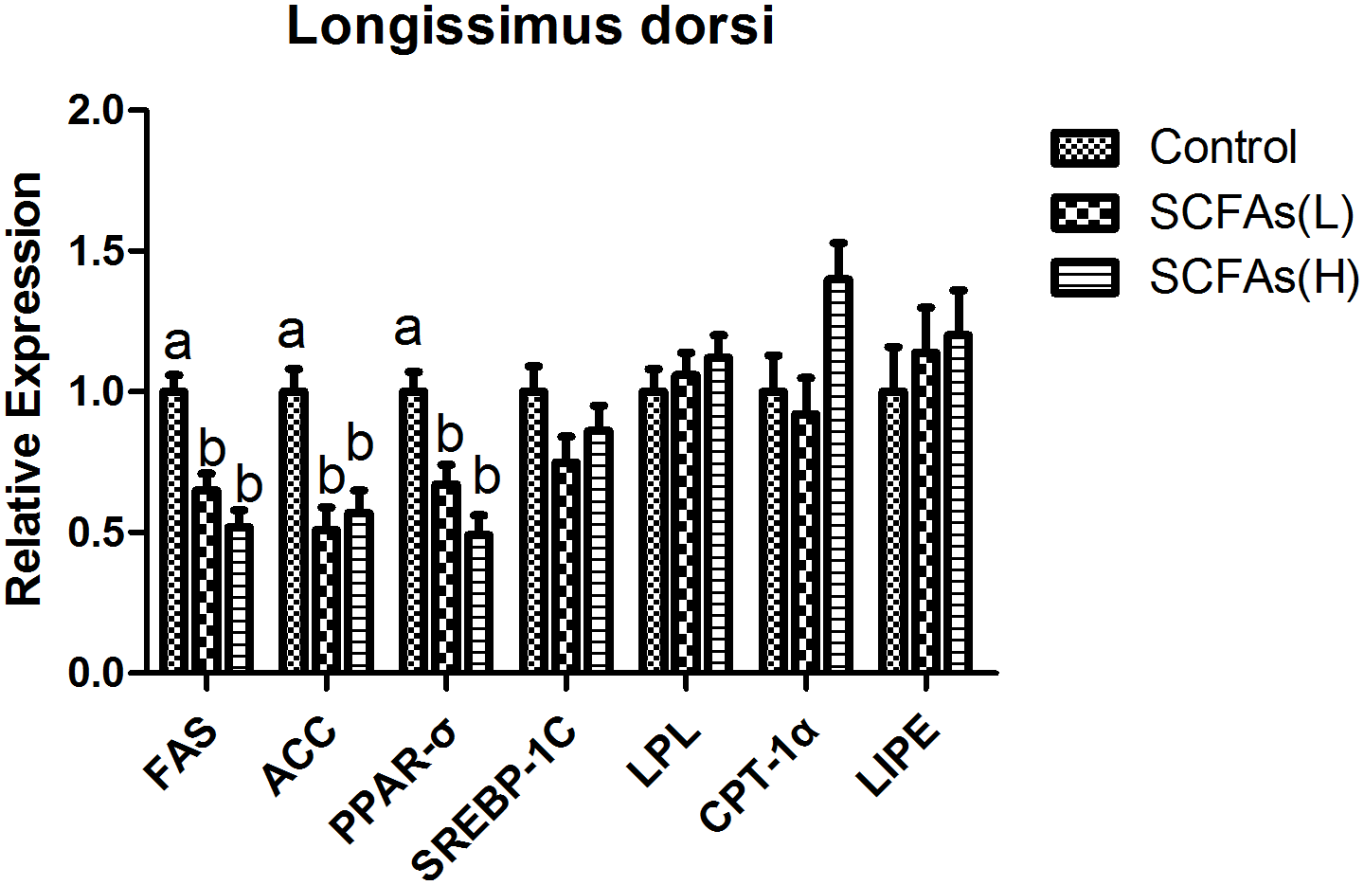
Effects of SCFAs on mRNA expressions for key factors related to lipid metabolism in longissimus dorsi over the experimental period of weaned pigs. SCFAs (L): acetic acid 20.04 mM, propionic acid 7.71 mM and butyric acid 4.89 mM; SCFAs (H) :acetic acid 40.08 mM, propionic acid 15.42 mM and butyric acid 9.78 mM. FAS fatty acid synthase; ACC acetyl-CoA carboxylase; PPAR peroxisome proliferator activated receptor; SREBP-1C sterol regulatory element binding protein 1C; LPL lipoprotein lipase; CPT-1α carnitine palmitoyltransferase-1α; LIPE lipase hormone- sensitive. ^a-b^Within a row, means without a common superscript differ (P<0.05).

**Fig 3:**
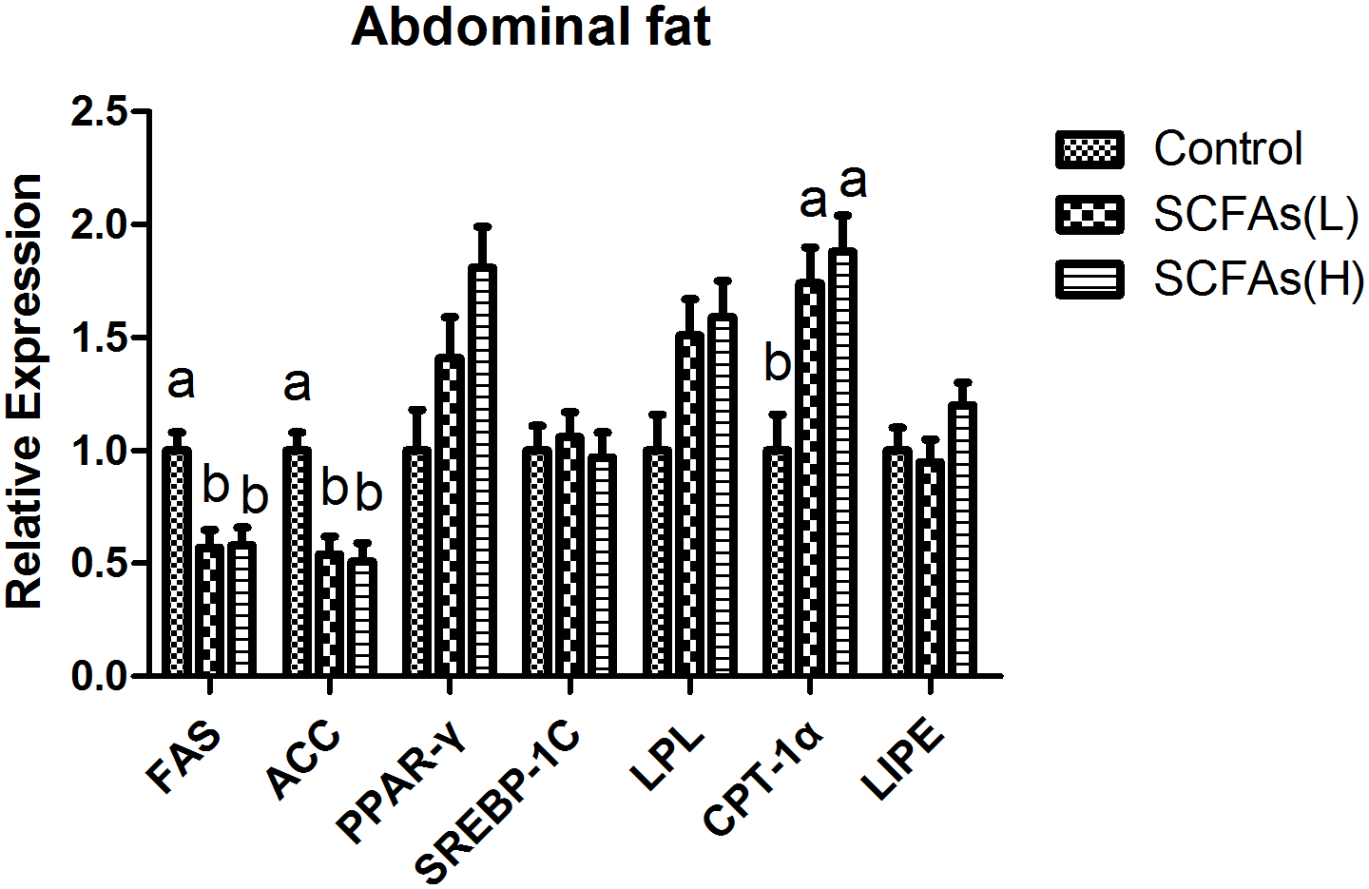
Effects of SCFAs on mRNA expressions for key factors related to lipid metabolism in abdominal fat over the experimental period of weaned pigs. SCFAs (L): acetic acid 20.04 mM, propionic acid 7.71 mM and butyric acid 4.89 mM; SCFAs (H) :acetic acid 40.08 mM, propionic acid 15.42 mM and butyric acid 9.78 mM. FAS fatty acid synthase; ACC acetyl-CoA carboxylase; PPAR peroxisome proliferator activated receptor; SREBP-1C sterol regulatory element binding protein 1C; LPL lipoprotein lipase; CPT-1α carnitine palmitoyltransferase-1α; LIPE lipase hormone- sensitive. ^a-b^Within a row, means without a common superscript differ (P<0.05).

Corresponding to lower total fat in liver, oral administration of SCFAs down-regulated the mRNA expressions of fatty acid synthetize (FAS) and sterol regulatory element binding protein 1c (SREBP-1c), and enhanced the mRNA expression of carnitine palmitoyltransferase-1α (CPT-1α) in liver (*P*<0.05, Fig 1). SCFAs also decreased FAS, acetyl-CoA carboxylase (ACC) and peroxisome proliferator activated receptor σ (PPARσ) mRNA levels in longissimus dorsi (*P*<0.05, Fig 2). In abdominal fat, SCFAs down-regulated the mRNA expressions of FAS and ACC, and enhanced the mRNA expression of CPT-1α (*P*<0.05, Fig 3).

## Discussion

Obesity has become one of the most serious health problems all over the world, which contributes greatly to a complex of symptoms that are called metabolic syndrome [18]. And SCFAs, the main products of dietary fiber by bacterial fermentation, have been shown to play important roles in the prevention and cure of metabolic syndrome [19]. According to previous experiment in which beet pulp was used as a dietary fiber source, the average concentrations of SCFAs in colon were as follows: acetic acid 40.08 mM, propionic acid 15.41 mM and butyric acid 9.78 mM (data not published). As the concentrations of SCFAs were chosen based on the colonic levels after feeding dietary fiber and oral SCFAs would be rapidly absorbed from the GI tract before reaching the colon, there may be differences between the positive effects of dietary fiber and oral SCFAs. The present study was mainly conducted to investigate the effects of oral SCFAs administration on the lipid metabolism of weaned pigs.

Previous researches demonstrated that SCFAs suppressed appetite through a central homeostatic mechanism [20]. This can be due to the fact that SCFAs stimulate the release of anorexigenic hormones peptide tyrosine tyrosine and glucagon-like peptide 1, and then delay gastric emptying [21]. In our study, oral administration of SCFAs significantly decreased ADFI and tended to decrease ADG, which were similar to the results of previous studies [22, 23].

Numerous changes in plasma lipid and lipoprotein are easily observed in obesity [24]. Indeed, obese individuals are often characterized by higher fasting plasma TG and TC compared with lean ones [25]. Here, we reported that oral administration of SCFAs decreased the concentrations of serum TG, TC and HDL-C, which were consistent with the results of previous studies about different SCFAs [26, 27]. These indicated that SCFAs might be beneficial to lowering some risk factors for obesity and diabetes.

Insulin is an important peptide hormone secreted by pancreatic beta cells, which modulates blood glucose level through inhibiting glycogen lysis. Thus, the balance between insulin production and insulin function is the key for maintaining glucose homeostasis [28]. Leptin, an adipose-derived hormone, can regulate energy homeostasis and several physiological processes (like feeding behavior and metabolic rate) [29], which is relative with inhibiting neuropeptide Y/agouti-related peptide neurons and activating POMC/cocaine and amphetamine-regulated transcript neurons [30]. In addition, leptin knock-out mice exhibited hyperphagia and obesity while leptin administration could reverse theses effects [31]. In this study, SCFAs decreased insulin level, and increased leptin level in serum, which were consistent with previous *in vivo* and *in vitro* studies [32, 33]. Therefore, it was possible that SCFAs administration decreased ADFI and lipid metabolism via regulating the levels of relative hormones.

Nonalcoholic fatty liver has been regarded as one of the most common liver diseases in the world, which is associated with obesity, insulin resistance and metabolic syndrome [34]. And the storage of TG in non-adipose tissues (like liver and skeletal muscle) that are known as ectopic fat deposition can lead to metabolic disorder and impaired organ function [35, 36]. In current study, we were pleased to find that oral administration of SCFAs decreased total fat as well as the concentrations of TG and TC in liver of pigs. Similarly, propionate could reduce intrahepatocellular lipid contents that met the diagnostic criteria of non-alcoholic fatty liver disease [27]. Therefore, SCFAs administration could be recommended as the therapy of nonalcoholic fatty liver.

SREBPs are lipid synthetic transcription factors that regulate the synthesis of cholesterol and fatty acid in liver [37, 38]. Previous study reported SCFAs suppressed cholesterol synthesis in rat liver and intestine [39]. We also found that liver TC level was lower in SCFAs groups compared with control group, which could be due to the fact that SCFAs down-regulated the mRNA expression of SREBP-1c in liver. FAS is an enzyme that catalyzes fatty acid synthesis while CPT-1α is a rate-limiting enzyme that participates in fatty acid oxidation [40]. ACC is an enzyme that regulates the metabolism of fatty acids, and its product, malonyl-CoA, act as building block for de novo fatty acid synthesis [41]. PPAR is a member of the nuclear receptor superfamily and regulates adipocyte differentiation and fat deposition [40]. In this study, SCFAs treatment down-regulated FAS mRNA level in liver, decreased FAS, ACC and PPARσ mRNA levels in longissimus dorsi, inhibited FAS and ACC mRNA levels in abdominal fat, and enhanced CPT-1α mRNA level in liver and abdominal fat. There were some similar changes of muscles and adipose tissues in previous studies [26, 42]. In addition, recent study showed that butyrate and other SCFAs increased the rate of lipolysis *in vitro* [43]. These suggested that SCFAs could reduce lipid accumulation by decreasing fatty acid synthesis and increasing lipolysis in different tissues.

## Conclusion

In summary, we demonstrated that SCFAs attenuated fat deposition in pigs via inhibiting feed intake, modulating endocrine and regulating lipid metabolism, which could provide some insights into the new therapies of obesity and nonalcoholic fatty liver.

## Supporting information

S1 File Minimal data set. (XLSX)
